# Implications of using whole genome sequencing to test unselected populations for high risk breast cancer genes: a modelling study

**DOI:** 10.1186/s13053-016-0052-7

**Published:** 2016-06-01

**Authors:** Charlotte Warren-Gash, Mark Kroese, Hilary Burton, Paul Pharoah

**Affiliations:** PHG Foundation, 2 Wort’s Causeway, Cambridge, CB1 8RN UK; Farr Institute of Health Informatics Research, University College London, 222 Euston Road, London, NW1 2DA UK; Centre for Cancer Genetic Epidemiology, Department of Public Health and Primary Care, University of Cambridge, Strangeways Research Laboratory, Wort’s Causeway, Cambridge, CB1 8RN UK

**Keywords:** Whole genome sequencing, Secondary findings, BRCA1, BRCA2, Unselected populations

## Abstract

**Background:**

The decision to test for high risk breast cancer gene mutations is traditionally based on risk scores derived from age, family and personal cancer history. Next generation sequencing technologies such as whole genome sequencing (WGS) make wider population testing more feasible. In the UK’s 100,000 Genomes Project, mutations in 16 genes including *BRCA1* and *BRCA2* are to be actively sought regardless of clinical presentation. The implications of deploying this approach at scale for patients and clinical services are unclear. In this study we aimed to model the effect of using WGS to test an unselected UK population for high risk *BRCA1* and *BRCA2* gene variants to inform the debate around approaches to secondary genomic findings.

**Methods:**

We modelled the test performance of WGS for identifying pathogenic *BRCA1* and *BRCA2* mutations in an unselected hypothetical population of 100,000 UK women, using published literature to derive model input parameters. We calculated analytic and clinical validity, described potential health outcomes and highlighted current areas of uncertainty. We also performed a sensitivity analysis in which we re-ran the model 100,000 times to investigate the effect of varying input parameters.

**Results:**

In our models WGS was predicted to identify correctly 93 pathogenic *BRCA1* mutations and 151 *BRCA2* mutations in 120 and 200 women respectively, resulting in an analytic sensitivity of 75.5-77.5 %. Of 244 women with identified pathogenic mutations, we estimated that 132 (range 121–198) would develop breast cancer, so could potentially be helped by intervention. We also predicted that breast cancer would occur in 41 women (range 36–62) incorrectly identified with no pathogenic mutations and in 12,460 women without *BRCA1* or *BRCA2* mutations. There was considerable uncertainty about the penetrance of mutations in people without a family history of disease and the appropriate threshold of absolute disease risk for clinical action, which impacts on judgements about the clinical utility of intervention.

**Conclusions:**

This simple model demonstrates the need for robust processes to support the testing for secondary genomic findings in unselected populations that acknowledge levels of uncertainty about the clinical validity and clinical utility of testing positive for a cancer risk gene.

## Background

Next generation sequencing technologies such as whole genome sequencing (WGS) have enhanced the speed and reduced the cost of seeking genetic mutations predisposing to conditions such as hereditary cancer syndromes [1]. There has, however, been intense debate about the appropriateness of using these technologies to screen individuals who do not meet traditional testing criteria. Arguments against population testing include uncertainty about the balance of benefit versus harm of interventions for high risk gene carriers without a strong family history of disease as well as other ethical, technical and cost concerns [2–6].

Internationally, clinical approaches to testing for and reporting secondary genomic findings, defined as findings that are actively sought by a practitioner but are not the primary target of investigation [7], vary widely. In the United Kingdom, the approach taken to secondary findings in the 100,000 Genomes Project–a flagship research project that aims to sequence 100,000 whole genomes from NHS patients by 2017 [8]–will act as a blueprint for future NHS practice. It is crucial therefore that the impact of using WGS for germline genetic testing on population health, clinical and laboratory services is appropriately considered.

In the 100,000 Genomes Project, deleterious alleles in 16 genes detected on whole genome analysis will be reported back to participants regardless of test indication [8]. These include the genes *BRCA1* and *BRCA2*, implicated in hereditary breast and ovarian cancer syndrome. In contrast, usual clinical practice is to undertake *BRCA1* and *BRCA2* testing based on results of risk scores calculated using factors such as age, family history and personal cancer history [9, 10]. Germline genetic testing for high risk cancer genes aims to provide the best possible estimate of an individual’s cancer risk to inform decisions about undergoing risk-lowering interventions. In the absence of a family history, the disease risk for mutations identified and therefore the clinical utility of testing, is likely to differ from that seen in multi-case families and may be poorly estimated.

In this study we aimed to model the likely outcomes of testing for medically-actionable gene mutations in unselected populations undergoing WGS, using the example of *BRCA1* and *BRCA2*. We considered the clinical validity of such testing and implications for individuals, laboratory and clinical services.

## Methods

### Model development

We built a simple model to calculate WGS test performance for identifying pathogenic *BRCA1* and *BRCA2* mutations in an unselected population of 100,000 UK women. Model input parameters were obtained from reviewing published literature on population prevalence of pathogenic *BRCA1* and *BRCA2* mutations and range and frequency of different mutation types including single nucleotide variants (SNVs), small insertions/deletions (indels) and copy number variants (CNVs). Where possible these were taken from studies in populations at low risk of breast cancer rather than multi-case families. We also used test performance literature for Illumina TruGenome Clinical Sequencing Services [11] and relevant laboratory standards [12, 13] to inform estimates of analytical validity–Table 1.

**Table 1:** Model input parameters (main analysis)

Input parameters for main model	Values for *BRCA1*	Values for *BRCA2*
Prevalence of pathogenic mutations in unselected population	0.0012	0.002
Theoretical population size	100000	100000
Proportion of pathogenic mutations that are small indels	0.54	0.69
Proportion of pathogenic mutations that are SNVs (nonsense, pathogenic missense, splice site)	0.36	0.21
Proportion of pathogenic mutations that are CNVs	0.1	0.1
Gene coverage using WGS	0.9941	0.9997
Sensitivity of WGS for small indels	0.8	0.8
Sensitivity of WGS for SNVs	0.97	0.97
Sensitivity of WGS of CNVs	0	0
Specificity of WGS for indels	1	1
Specificity of WGS for SNVs	1	1
Specificity of WGS for CNVs	1	1

### Analytic validity calculations

Calculations shown below for *BRCA1* were repeated for *BRCA2*. We assumed that variants of uncertain significance (VUS) were not reported back to patients in line with common practice [14].

- *True positives* = prevalence of pathogenic *BRCA1* mutations x population size x (proportion of *BRCA1* mutations that are small indels x sensitivity of WGS for detecting small indels + proportion of *BRCA1* mutations that are SNVs x sensitivity of WGS for detecting SNVs + proportion of *BRCA1* mutations that are CNVs x sensitivity of WGS for detecting CNVs) x horizontal gene coverage of WGS for *BRCA1* *
- *False negatives* = prevalence of pathogenic *BRCA1* mutations x population size–*true positives*
- *False positives* = in main model assumed to be 0 after a confirmatory step*. In sensitivity analysis we modelled the effect of an extra 0-5 % of *false positive* results in addition to *true positive* results
- *True negatives* (*including VUS*) = population size-prevalence x population size-*false positives*

Test performance measures of WGS for detecting pathogenic mutations were calculated as follows:

- *Analytical sensitivity* = *True positives* detected/Total with a true pathogenic mutation
- *Analytical specificity* = *True negatives* detected/Total without a pathogenic mutation
- *Analytical positive predictive value* = *True positives* detected/Total with a variant on testing
- *Analytical negative predictive value* = *True negatives* detected/Total without a variant on testing

*Note in line with usual practice for next generation sequencing we assumed that any positive results would be confirmed by an independent test from a new DNA dilution or a secondary test e.g. a SNP assay [13]. It is not usual practice to confirm all negative findings but we assumed for the model that reporting standards for negative findings were met [13].

### Sensitivity analysis

**Fig. 1:**
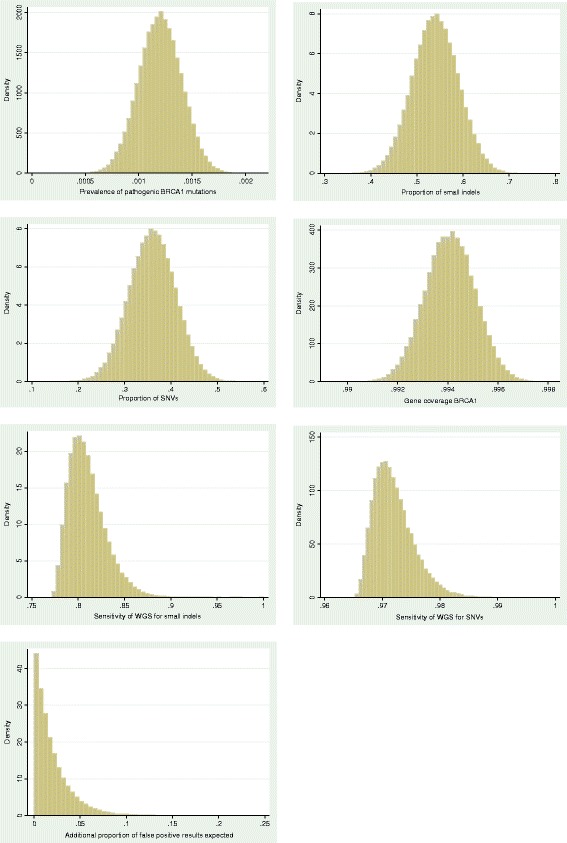
Distribution of model input parameters used for sensitivity analysis using example of *BRCA1*

We also performed a sensitivity analysis to investigate the effect of varying model input parameters. The model was rerun 100,000 times with model input parameters being randomly selected from defined likely distributions using Stata’s random number generator function. The proportion of pathogenic mutations due to CNVs was assumed to be fixed at 0.1, but the proportion of small indels and SNVs varied according to an underlying normal distribution. Sensitivity of WGS for detecting CNVs was fixed at 0, based on current test performance literature, but sensitivity for detecting SNVs and small indels was selected from an underlying gamma distribution. We also assumed that false positives would occur at a rate of less than 10 % of the number of true positives, but heavily skewed towards 0. Calculations for true positives, false negatives and true negatives remained the same as for the main model. Underlying distributions for model input parameters are shown in Fig. 1.

### Assessing clinical validity

We assessed the performance of WGS for detecting future risk of breast cancer by applying population penetrance estimates from published literature to modelled numbers of women with each test outcome–true positives, false positives, true negatives and false negatives. We did not evaluate the clinical utility of testing for pathogenic *BRCA1* and *BRCA2* mutations because such testing is well-established internationally in the context of multi-case families. Evaluation of the complex ethical, legal and social implications was outside the scope of this paper.

## Results

### Prevalence and types of *BRCA1* and *BRCA2* mutations

In an unselected UK population we initially assumed a carrier frequency of 0.12 % for pathogenic *BRCA1* mutations and 0.2 % for *BRCA2* mutations [15], which equated to 120 *BRCA1* and 200 *BRCA2* mutations in 100,000 unselected women. In our main model we also assumed that pathogenic *BRCA1* mutations comprised 54 % small indels, 36 % SNVs and 10 % CNVs; corresponding figures for pathogenic *BRCA2* mutations were 69 % small indels, 21 % SNVs and 10 % CNVs. This was based on findings that around 88-90 % of pathogenic *BRCA1* and *BRCA2* mutations are due to missense, nonsense and splice site mutations as well as small indels, with the remaining 10-12 % due to large rearrangements/duplications [16]. A recent population series of 2,222 ovarian cancer cases and 1,528 controls characterised the spectrum of mutations further: 85 *BRCA1* mutations were detected, comprising 51 frameshift indels and 34 missense, nonsense or splice site mutations; there were also 98 *BRCA2* mutations, made up of 75 frameshift indels and 23 missense, nonsense or splice site mutations [17].

### Penetrance of *BRCA1* and *BRCA2* mutations

In our main model we used penetrance figures of 59 % for *BRCA1* and 51 % for *BRCA2* based on average cumulative risk of female breast cancer modelled over all possible modifiers for carriers born after 1950 [15], but also included ranges of 57-71 % for *BRCA1* [18–20] and 45–87.5 % [21, 22] for *BRCA2* based on other literature findings. The penetrance of these mutations for breast cancer varies with age at testing, family history and mutation type, depending on a complex interplay between the genetic variant and other environmental and genetic modifiers. Consequently, published estimates of penetrance vary, reflecting both the effect of differing risk modifiers and methods of ascertainment of mutation carriers. Estimates from studies based on multi-case families are typically higher than those based on unselected breast cancer cases. There are no empirical estimates of the average penetrance for an unselected mutation carrier. However, estimates of the average risk have been derived from complex segregation analyses using data from both multi-case families and breast cancer case series by explicitly modelling the contribution of modifiers [15], which we considered the most appropriate estimates for our study.

### Factors affecting WGS performance

Analytical validity of WGS for identifying and classifying genetic variants correctly depends on a range of factors including depth of coverage as well as technical accuracy for detecting different mutation types. In our model we assumed an adequate read depth but recognised that in practice the minimum depth of coverage varies depending upon the required sensitivity of the assay, the sequencing method and the type of mutation detected and should be established during the test validation process [13]. Algorithms in current clinical use allow >97 % of SNVs and >80 % of deletions to be identified by WGS but may be unable to detect larger insertion/deletion events of >7 bp, translocations, trinucleotide repeats or CNVs [11], although this area is developing rapidly. An additional factor is horizontal coverage across the genome, which is typically > 95 % for WGS, but reported as 99.41 % in the region of *BRCA1* and 99.97 % for *BRCA2* [11].

### Test performance of WGS for detecting *BRCA1* and *BRCA2* pathogenic mutations

In our main model WGS successfully detected 93 women out of 120 with pathogenic *BRCA1* variants and 151 women out of 200 with pathogenic *BRCA2* variants in a hypothetical UK population of 100,000 women. This gave an analytic sensitivity of 77.5 % for *BRCA1* variants and 75.5 % for *BRCA2*. Specificity was 100 %, with 100 % PPV and 99.9 % NPV for both genes. Sensitivity analysis gave similar results–Table 2.

**Table 2:** Numbers of pathogenic *BRCA1* and *BRCA2* mutations detected in an unselected population of 100 000 UK women using WGS

*BRCA1* main model	Has gene variant?				*BRCA2* main model	Has gene variant?			
Variant detected by WGS?	Yes	No	Total		Variant detected by WGS?	Yes	No	Total	
Yes	93	0	93		Yes	151	0	151	
No	27	99 880	99 907		No	49	99 800	99 849	
Total	120	99 880	100 000		Total	200	99 800	100 000	
*BRCA1* sensitivity analysis	Mean (SD)	Median (IQR)	Min	Max	*BRCA2* sensitivity analysis	Mean (SD)	Median (IQR)	Min	Max
True positives	94 (15.8)	94 (83–105)	26	175	True positives	153 (23.0)	153 (137–168)	49	245
False positives	2 (1.9)	1 (0–3)	0	26	False positives	3 (3.1)	2 (1–4)	0	37
False negatives	26 (4.7)	26 (23–29)	7	48	False negatives	48 (7.8)	47 (42–53)	16	88
True negatives	99 878 (20.4)	99 878 (99 864–99 891)	99 777	99 967	True negatives	99 797 (30.5)	99 796 (99 776–99 817)	99 672	99 934

### Clinical validity

**Fig. 2:**
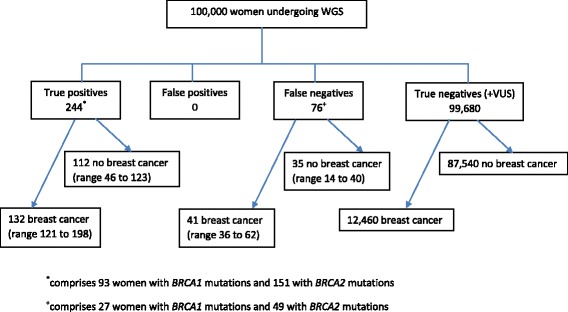
Flowchart of expected breast cancer incidence in unselected women undergoing WGS

The performance of WGS for predicting future risk of breast cancer depends both on test performance and on the association between genotype and disease. In our model, WGS correctly identified 132 women (range 121–198) with a pathogenic *BRCA1* or *BRCA2* mutation who would develop breast cancer. We also estimated that breast cancer would occur in 41 women (range 36–62) incorrectly identified with no pathogenic mutations and in 12,460 women who truly had no mutation, based on a background lifetime risk of female breast cancer of 12.5 % in the UK [23] Fig. 2.

### Potential health outcomes

#### *Scenario 1 Analytic true positive women* = 244/100,000

Positive health outcomes include reductions in breast cancer incidence and mortality for the 132 women predicted to develop breast cancer and their family members. The 112 other analytic true positive women would have the same preventive options but receive no benefit. Potential negative consequences include physical and psychological harms of chemoprevention or prophylactic surgery.

#### *Scenario 2 Analytic false positive women* = 0/100,000

There are no positive health outcomes for this group. All negative health outcomes identified for true positive women would apply, although numbers affected are likely to be extremely small.

#### *Scenario 3*: *Analytic false negative women* = 76/100,000

This group will have no positive health outcomes. There may be false reassurance associated with receiving a negative result. Opportunities to prevent breast cancer will be missed.

#### *Scenarios 4 Analytic true negatives* (*includes VUS*) = 99 680/100,000

There are no direct positive or negative health outcomes for this group, although without careful consent and clear clinical feedback some may be falsely reassured that they are not at risk of breast cancer which might affect subsequent health behaviour.

## Discussion

Using the example of pathogenic *BRCA1* and *BRCA2* mutations we demonstrate the type of process that should be undertaken when considering likely outcomes of testing for secondary genomic findings in unselected populations. We estimated that WGS would detect 75.5-77.5 % of pathogenic *BRCA1* and *BRCA2* mutations, with the majority of undetected mutations comprising CNVs. This is well below the 95 % sensitivity threshold recommended for clinical genetic diagnostic tests [13]. In a hypothetical UK population of 100,000 women, this would result in 244 identified for further interventions, potentially preventing around 132 cases of breast cancer. This would also result in unnecessary inteventions for 112 women with mutations predicted not to develop cancer, although this is in line with current practice [10]. We note that outside the context of WGS, routine *BRCA1* and *BRCA2* testing is neither recommended nor advocated at population level [9, 10, 24].

Key areas of uncertainty include limitations to current knowledge of the prevalence, spectrum and penetrance of pathogenic mutations associated with a variety of hereditary diseases, including those currently recommended for routine examination on WGS by Genomics England. In hereditary breast cancer, estimates of penetrance have frequently been derived from studies conducted in multi-case families as population estimates do not exist, and it may not be appropriate to apply such estimates to unselected populations undergoing WGS. For mutations in other genes conferring a lower risk of disease, there is uncertainty about the threshold for clinical action and thus the level of absolute disease risk at which secondary findings should be fed back.

Care pathway factors should also be considered when implementing this approach. Currently WGS has sub-optimal sensitivity for detecting certain types of mutation, in particular CNVs and some indels. Concerns have been raised about potential inconsistencies between laboratories in assuring quality of data generated by WGS and its interpretation [25]. The risk of false positives, although low, would be increased if laboratories did not undertake a confirmatory step. The number of pre-symptomatic mutation carriers identified across a range of genes tested for secondary findings is unknown so there is a lack of assurance that clinical services will be able to manage the extra work volume generated. For *BRCA1* and *BRCA2* mutation carriers this would include enhanced radiologic surveillance, chemoprophylaxis and/or prophylactic surgery to mitigate risk. In addition the cost effectiveness of such interventions is uncertain although recent data suggest that screening of generally healthy individuals using next generation sequencing may not currently be cost effective [26].

Globally there is divergent policy around secondary genomic findings: in 2013 the American College of Medical and Genetics and Genomics (ACMG) controversially recommended routine examination of 56 potentially actionable genes and types of variants whenever clinical exome or genome sequencing is undertaken [27] (although an opt out clause has since been added) [28]; the European Society of Human Genetics in contrast suggests using a targeted testing or reporting strategy where possible to minimise the risk of genetrating unsolicited findings [29]. In the UK the 16 genes recommended for routine examination in the 100,000 Genomes Project are based on the ACMG list plus ‘subsequent expert opinion’ [7]. In unselected populations, however, it is unclear how well this approach to secondary genomic findings allows quantification of an individual’s absolute disease risk, which is essential to making valid judgements about risks and benefits of clinical intervention.

## Conclusions

In summary, we use a simple model to highlight issues that hinder the utility of actively seeking secondary findings using WGS, even for relatively well-characterised genes. Applying this method to other gene-disease combinations is likely to reveal further gaps. It is therefore imperative that robust processes are in place for managing and understanding these complex data and appreciating the levels of uncertainty around clinical validity and clinical utility of testing positive for a cancer risk gene. Detailed evaluation of developing practice and research will be essential to enable effective clinical implementation of this approach to secondary genomic findings in unselected populations.
